# Interocular Shift of Visual Attention Enhances Stereopsis and Visual Acuities of Anisometropic Amblyopes beyond the Critical Period of Visual Development: A Novel Approach

**DOI:** 10.1155/2014/615213

**Published:** 2014-12-15

**Authors:** Liwen Huang, Xinghuai Sun, Gang Luo, Shuai Liu, Rui Liu, Behzad Mansouri, Vicky wing lai Wong, Wen Wen, Hong Liu, Ai-Hou Wang

**Affiliations:** ^1^Department of Ophthalmology, Eye & ENT Hospital, Fudan University, 83 FenYang Road, Shanghai 200031, China; ^2^Key Laboratory of Myopia, Ministry of Health, Eye & ENT Hospital, Fudan University, 83 FenYang Road, Shanghai 200031, China; ^3^Schepens Eye Research Institute, Massachusetts Eye and Ear, Department of Ophthalmology, Harvard Medical School, Boston, MA 02135, USA; ^4^Department of Internal Medicine, Section of Neurology, Room GF328, Health Sciences Centre, University of Manitoba, 820 Sherbrook Street, Winnipeg MB, Canada R3T 2N2; ^5^Department of Ophthalmology, National Taiwan University Hospital, 7 ZhongShan S Road, Taipei 10002, Taiwan

## Abstract

*Aims.* Increasing evidence shows that imbalanced suppressive drive prior to binocular combination may be the key factor in amblyopia. We described a novel binocular approach, interocular shift of visual attention (ISVA), for treatment of amblyopia in adult patients. *Methods.* Visual stimuli were presented anaglyphically on a computer screen. A square target resembling Landolt C had 2 openings, one in red and one in cyan color. Through blue-red goggles, each eye could only see one of the two openings. The patient was required to report the location of the opening presented to the amblyopic eye. It started at an opening size of 800 sec of arc, went up and down in 160 sec of arc step, and stopped when reaching the 5th reversals. Ten patients with anisometropic amblyopia older than age 14 (average age: 26.7) were recruited and received ISVA treatment for 6 weeks, with 2 training sessions per day. *Results.* Both Titmus stereopsis (*z* = −2.809, *P* = 0.005) and Random-dot stereopsis (*z* = −2.317, *P* = 0.018) were significantly improved. Average improvement in best corrected visual acuity (BCVA) was 0.74 line (*t* = 5.842, *P* < 0.001). *Conclusions.* The ISVA treatment may be effective in treating amblyopia and restoring stereoscopic function.

## 1. Introduction

Amblyopia is a developmental disorder associated with early abnormal visual experience that disrupts neuronal circuitry in the visual cortex and results in abnormal vision. In the past, the patients with amblyopia beyond the age of sensitive period of brain development generally received no treatments because of the long believed notion that, beyond the critical period, the neural network structure is not flexible and lacks plasticity [1]. Therefore, the traditional treatments for amblyopia, for example, patching or penalizing the fellow eye, have been limited to children at critical period age only (i.e., before 10 years old).

In the recent years, there has been accumulating evidence showing the existence of neural plasticity in the mature human visual system [2]. Plasticity in adults with amblyopia is also dramatically evident in the reports of amblyopic patients whose visual acuity spontaneously improved in the wake of visual loss due to macular degeneration in the fellow eye [3, 4]. Adults with amblyopia can improve their perceptual performance and visual acuity through extensive practice on a challenging visual task [2]. Meanwhile, animal study showed environmental enrichment in adult amblyopic rats restored normal visual acuity and ocular dominance [5].

Recently, it has been shown that the monocular vision in adults with amblyopia can be improved after a 10 min application of repetitive transcranial magnetic stimulation to the visual cortex, suggesting that a significant part of the monocular vision loss might be suppressive in nature [6]. These findings are consistent with the notion that the connections from the amblyopic eye may be suppressed during their lifetime rather than being destroyed. The predominant theory suggests that amblyopia results from a mismatch between the images of two eyes; one eye is favored and the other eye is suppressed [7]. Animal studies [8] and clinical evidence [9] also have indicated that suppression is a key mechanism that causes amblyopia. The imbalanced suppressive drive prior to binocular combination may be the key factor in amblyopia and thus is different from those that are caused by monocular loss of function.

This perspective allows us to approach the treatment of amblyopia in a radically different way, that is, weakening the suppression. It is known that information from an amblyopic eye can be strongly suppressed when both eyes are open [10]. To reduce the suppression, it is necessary to have the presence of such suppression, so it may not be ideal to restore the vision in the amblyopic eye using only monocular methods. We argue that taking a binocular approach to amblyopia treatment may offer a more principled and effective option [11, 12].

Recently, there have been a few reports on binocularly focused models of treatment of amblyopia in adults [11, 13]. In single-noise binocular training created by Hess et al. [11] stimulus are shown to the amblyopic eye at high contrast, while the contrast of the stimuli shown to the nonamblyopic eye is decreased. Here, we designed a novel approach to shift interocular visual attention under binocular viewing condition, in which the same contrast of stimuli is shown to the amblyopic eye and nonamblyopic eye and another feature is to fully use patient's attention. We hypothesized that attention shift may improve the vision in the amblyopic eye as well. This active procedure rather mimicked the binocular viewing situation in amblyopia rendering binocular rivalry.

There are two major categories of monocular amblyopia: anisometropic and strabismic. The causing mechanisms of strabismic and anisometropic amblyopia are different. In anisometropic amblyopia, the amblyopic eye does not see the high spatial frequencies, while the low spatial frequency contour falls on the corresponding area in both eyes [14]. The causing mechanism includes both suppression and deprivation of the amblyopic eye [15]. The viewing condition of strabismic amblyopia is even worse that all spatial frequencies of the images fall on noncorresponding areas in the two eyes, and binocular suppression covers all spatial frequencies to avoid diplopia. Therefore, antisuppression treatment for strabismic amblyopia may lead to diplopia before the eyes are aligned optically or surgically or if the sensory fusion after improvement of binocularity and vision in amblyopic eye is not sufficient to prevent double vision [2]. So, only patients with anisometropic amblyopia were enrolled in this study.

## 2. Methods

### 2.1. Patients and Measurements

The inclusion criteria were (1) age over critical period age, (2) anisometropic amblyopia with binocular difference of best corrected visual acuity (BCVA) equal to or more than two lines, and (3) BCVA of the amblyopic eye equal to or worse than 0.8.

Ten patients (age 14–34 years) were recruited from July 2011 to September 2012 in our clinic. All of the patients in the trial underwent initial ocular examination that included assessment of the BCVA before the training; lack of improvement was confirmed after wearing glasses for 3 months. Titmus stereopsis, Random-dot stereopsis, and BCVA were measured before the training and 6 weeks after the training. All pre- and postvisual examinations were compiled by the same specially trained optometrist for consistency in visual acuities and stereoscopic measurements.

This study was approved by the Institutional Review Board of Eye and ENT Hospital of Fudan University. All participants gave written informed consent in accordance with the Declaration of Helsinki (2008).

### 2.2. Treatment Protocol

The interocular shift of visual attention (ISVA) training consisted of a pair of reusable glasses with two colored filters and software for use on a personal computer. During the training, patients were instructed to maintain a constant distance between the eyes and the screen of computer. The software was designed to be engaging and interactive, which was designed by one of the authors (AHW). The patients were given a copy of this program to perform self-training at home. They were asked to have 2 training sessions each day, each session lasting for 15 to 30 minutes. The patients were followed up at 6 weeks after the training began.

**Figure 1:**
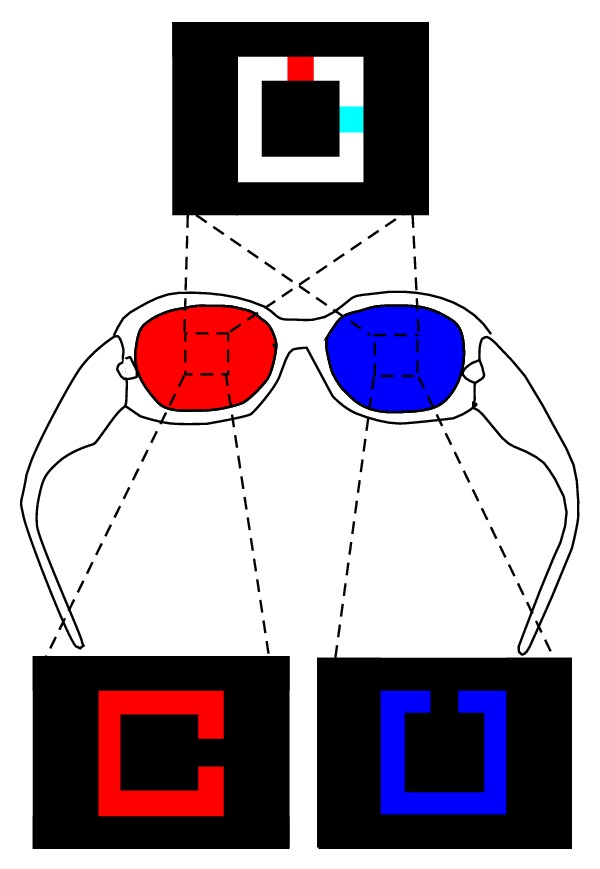
Stimulus paradigm of the interocular shift attention. The top of this diagram shows the training visual stimulus seeing through the anaglyphic glasses (shown in the middle); two eyes see different images (shown at the bottom). The task is to detect the opening direction of the image seen by the amblyopic eye, using a 4-alternative forced-choice (4-AFC) training paradigm.

**Figure 2:**
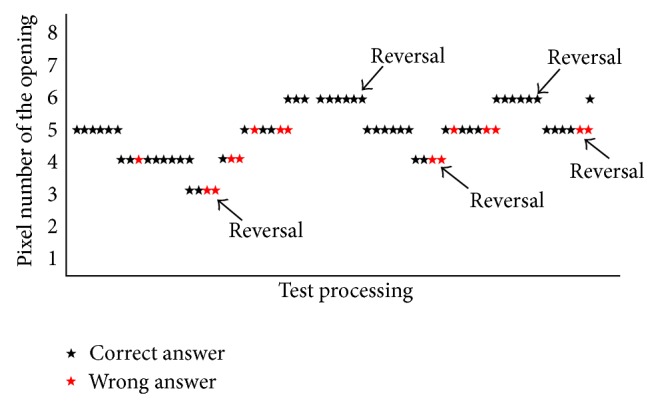
An example of the 6-down-2-up staircase paradigm. It started at an opening size of 5 pixels, went up and down in 1 pixel step, and stopped when reaching the 5th reversals along the test procedure. The logarithms of the visual angles at these 5 reversals were averaged to give an estimate of visual acuity at this training. On a 19-inch 16 : 9 screen set at 1366 × 768 resolution, one pixel was equivalent to 160 sec of arc when viewed at 40 cm test distance.

The visual stimuli for ISVA training were presented anaglyphically (Figure 1). The square target mimicked Landolt C. The size of the opening was one-fifth of the whole target. Visual angle of the target could be changed as shown in the staircase paradigm (Figure 2). There were 2 openings: one in red color and one in cyan color. Through blue-red goggles, the red opening was seen only by the eye wearing blue lens and the cyan opening was seen only by the eye wearing red lens. The task was to push one of four arrow keys to indicate the opening direction that was seen by the amblyopic eye. The opening direction was a random 4-alternative forced-choice (4-AFC) each time.

During training, the fellow eye was first occluded to make sure the patients focused their attention on the image from the amblyopic eye and saw the opening. The patients selected the direction of cyan opening if the amblyopic eye was fitted with the red lens or selected the direction of red opening if the amblyopic eye was fitted with the blue lens. Take, for example, a patient with his/her amblyopic eye fitted with the red lens. After the amblyopic eye saw the cyan opening, the occluder was removed. The opening was not stable due to binocular rivalry. The patient was asked to keep attention on the cyan opening; meanwhile, his/her good eye could also see the red opening through the blue lens. The red C and the cyan C seen by the patient fell on the corresponding areas in both eyes and could be fused, like in normal subject. The patient fused these two Cs into a white square ring with the red and the blue openings.

The training used a 6-down-2-up staircase paradigm (Figure 2). It started at an opening size of 5 pixels, went up and down in 1 pixel step, and stopped when reaching the 5th reversals along the test procedure. The logarithms of the visual angles at these 5 reversals were averaged to give an estimate of visual acuity at this training. On a 19-inch 16 : 9 screen set at 1366 × 768 resolution, one pixel was equivalent to 160 sec of arc when viewed at 40 cm test distance. Six consecutive right answers would lead to a smaller visual angle, while two consecutive wrong answers would lead to a larger visual angle in each step. We increased the number of correct answers in the process to emphasize its training purpose rather than threshold approaching. The task started at an opening size of 800 sec of arc, went up and down in 160 sec of step, and stopped when it reached the 5th reversals. The logarithms of the visual angles at these 5th reversals were averaged to give an estimate of visual acuity at this training.

### 2.3. Statistics

Due to the discrete data, differences between pre- and posttraining stereopsis (Titmus stereopsis and Random-dot stereopsis) were assessed using Wilcoxon sign rank test. Differences between pre- and posttraining BCVA were assessed using paired *t*-test. Discrete data were presented as median and range, while continuous data were presented as mean and range. All statistical assessments were two-tailed and were considered significant at the 0.05 level. Statistical analyses were performed using SPSS 15.0 statistics software (SPSS Inc, Chicago, IL).

## 3. Results

**Figure 3:**
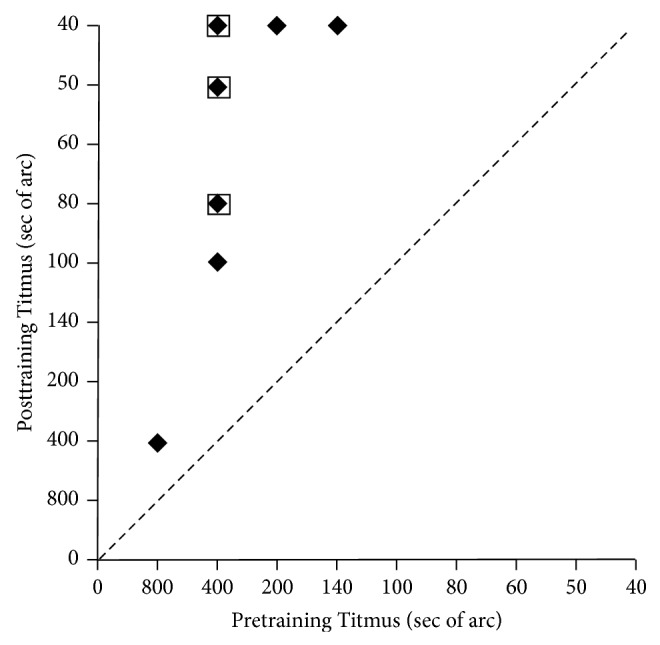
Data of Titmus stereopsis before and after the training. The* x*-axis represents the stereopsis of the subjects before the treatment and the* y*-axis represents the stereopsis of subjects after the treatment. Stereopsis of Titmus was significantly improved after the treatment (*P* = 0.005). A square outline represents two overlapping data points.

**Figure 4:**
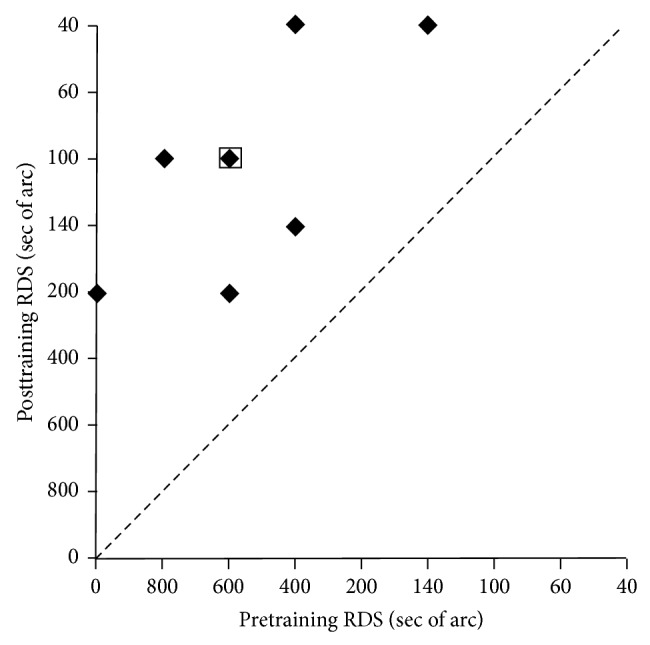
Random-dot stereogram data before and after the training. The* x*-axis represents the RDS of the subjects before the treatment and the* y*-axis represents the RDS of the subjects after the treatment. Stereopsis of Random-dot stereogram was statistically significantly improved (*P* = 0.018). RDS: Random-dot stereograph. A square outline represents two overlapping data points.

ISVA training led to significantly improvment in stereopsis. As shown in Table 1, ten amblyopic patients participated in this study, 6 males and 4 females. The average age of these patients was 26.7 years (range, 14 to 34 years). Median thresholds of Titmus stereopsis and Random-dot stereopsis were 400 sec (range, 140 to 800 sec) and 500 sec (range, 140 to 800 sec) before the training. Posttraining measurements were taken at 6 weeks. The same measurement procedures were used for pre- and posttraining tests. Median thresholds of Titmus stereopsis and Random-dot stereopsis were 50 sec (range, 40 to 400 sec) and 120 sec (range, 40 to 600 sec) after the training. Figures 3 and 4 showed the comparison of these measurements before and after the training. Notably, 2 patients who had no measurable stereoscopic depth perception in Random-dot stereopsis before the training acquired 200 sec and 600 sec after the training, respectively. Both Titmus stereopsis (Wilcoxon sign rank test: *z* = −2.809, *P* = 0.005) and Random-dot stereopsis (Wilcoxon sign rank test: *z* = −2.317, *P* = 0.018) were significantly improved after ISVA training.

### 3.1. Visual Acuity Was Improved after ISVA Training

**Figure 5:**
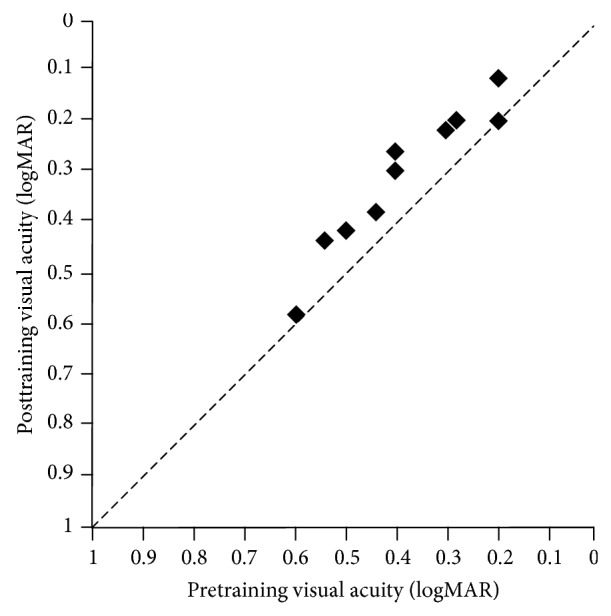
Visual acuity in amblyopic eye before and after the training. The* x*-axis represents the visual acuity (logMAR) of the subjects before the treatment and the* y*-axis represents the visual acuity (logMAR) of the subjects after the treatment. Results indicate improvement in visual acuities of the amblyopic eye after the ISVA training (*P* = 0.000).

We also measured BCVA before and after the training. As seen in Figure 5 and Table 1, in amblyopic eyes, average BCVA before the training was 0.39 ± 0.14, and average BCVA after the training was 0.31 ± 0.14. Improvement in BCVA after ISVA training was 0.74 line (paired* t*-test: factor of 1, *t*8 = 5.842, *P* < 0.001). In the follow eyes, pretraining value was −0.05 ± 0.07 and posttraining value was −0.05 ± 0.07. The BCVA had no changes (paired *t*-test: factor of 1, *t*8 = 0.218, *P* = 0.832).

## 4. Discussion

The mainstay of amblyopia treatment nowadays is occlusion and/or penalization with atropine or over-plus lens to the fellow eye. All these approaches rely on a passive procedure to make the fellow eye see less and enhance the vision in the amblyopic eye. Here, we used ISVA training, a novel binocular approach, for anisometropic amblyopes and demonstrated that this active procedure could significantly improve visual acuity in the amblyopic eye and stereopsis by rendering binocular competition.

One main feature of our treatment is to fully use patient's attention. Patients learned to use their visual attention by forcing the amblyopic eye to actively search for repeatable and calibrated targets of certain arc minutes within their visual field. Visual attention is taken as a trivial matter in our daily life. When you focus your attention on a person within your visual field, despite the fact that you do not fixate on him/her, you get more information of his/her posture or expression and so forth, compared with when you do not focus your attention on him/her [16]. There must be some changes in the brain before and after focusing the attention [17].

Recent neurophysiological studies have demonstrated that if a preferred stimulus and a nonpreferred stimulus are presented in one neuron's receptive field, cell's responsiveness depends on the attentional state. Attending to the preferred stimulus increases the cell's firing rate, whereas attending to the nonpreferred stimulus attenuates it [18]. This finding suggests that attention shifts the receptive field of the cell at the attended location [19, 20]. Thus, neurons with receptive fields at that location either remain active or become more active, while the others are suppressed.

Visual attention in monocular viewing condition manifests as that visual acuity increases at attended location and decreases at unattended location within the visual field [21]. The patients with monocular amblyopia naturally fixate with their fellow eyes. Along similar rationale, we hypothesize that ISVA might increase the visual acuity of the amblyopic eyes, either transiently or permanently. Our study confirmed this hypothesis.

Another important feature of our treatment is the use of binocular stimulus. Although monocular treatments that excite the weak eye alone is effective [2, 22], Ooi et al. [13], Xu et al. [23], and Li et al. [24] have shown that binocular training is more effective than monocular training to excite the amblyopic eye, while completely inhibiting the strong eye's perception to recalibrate the interocular balance of excitatory and inhibitory interactions. They indicated that adult amblyopes trained with a binocular protocol gained improvements in visual acuity and stereopsis [13, 23, 24]. To et al. [25] trained patients using an iPod video game, whereby stimuli elements were presented dichoptically, with lower contrast stimuli being presented to the fellow eye to counteract the suppression and allow for binocular combination. The improved visual acuity and stereopsis results were significantly greater in groups that were trained by binocular therapy than monocular therapy. The common feature between our study and previous two studies [13, 25] is the use of binocular viewing conditions. All three studies showed significant treatment effects. In our binocular training paradigm, visual attention is highly demanded in the amblyopic eye, which helps to recalibrate the interocular balance of excitatory and inhibitory interactions [26]. The procedure of applying proactive attention is the very procedure of modulating the excitatory and inhibitory circuits.

Currently, in most available video games for treatment of amblyopia, the stimuli elements were presented separately to each eye and lower contrast stimulus was presented to the fellow eye to enhance the visibility for amblyopic eye to counteract suppression and allow for binocular combination. What was unique in our design was that the ISVA in our experiment was an active process. Patients learned to use their visual attention by forcing the amblyopic eye to actively search for repeatable and calibrated targets of certain arc minutes within their visual field. The image seen by the fellow eye was kept in a good visibility during the whole training course. Cortical neurons with receptive fields at that location either remain active or become more active, while the others are suppressed. It was interesting that this equal visibility of the images in both eyes in our task resembled the condition as in the course of binocular rivalry that caused the amblyopia in the first place.

Binocular rivalry has been shown to produce a characteristic counter-phase pattern in the signal from the two eyes. As the image in one eye becomes dominant, its cortical signal strengthens and the signal corresponding to the other eye weakens [19]. Recent studies [20] have shown strong training effect by using rivalry stimuli and directing patient's attention to the amblyopic eye image. These studies suggest that the rivalry suppression of the strong eye not only enhances the weak eye's excitatory signal (a “push” effect), but also strengths the amblyopic eye's inhibition to the strong eye (a “pull” effect). Our stimulus paradigm may also have the same effect since the two eyes are seeing images in different colors and may rival over time. The initial cue (paying attention to the notch seen by the amblyopic eye) works in the same way as in the binocular rivalry study [20], and thus our training procedure may also use the same “push-pull” neural mechanism. During our experiment, the patients were engaged in finding the targets using their amblyopic eyes, which was so challenging to their visual system that the training was intensive and active, which may lead to a more efficient result.

Limitations to our experiment include our small sample size of ten patients. Improvements to this experiment could be a larger sample size, a more even distribution of patient ages and their severity of amblyopia, and longer training sessions in the future. Duration of the study can be lengthened by increasing the patients' motivation, which can be achieved by informing the patient upfront of potential acuity and binocular improvements translating to an enhanced daily life in driving, working, and social activities.

## 5. Conclusion

This is the first study for this novel device to be used in the treatment of amblyopia and demonstrates an improvement in visual acuity and stereopsis. More patient samples in a sham controlled randomized clinical trial are required to verify its true effectiveness and place in future management of amblyopia.

## Figures and Tables

**Table 1:** Ten patients' information before and after training by interocular shift of visual attention.

Patient number	Age (year)	Visual acuity of the amblyopic eye (logMAR)		Titmus stereopsis (sec of arc)		Yans Random-dot training (sec of arc)	
		Before training	After training	Before training	After training	Before training	After training
1	25	0.60	0.58	400	50	—	200
2	34	0.30	0.22	400	40	600	100
3	14	0.28	0.20	200	40	800	100
4	26	0.40	0.26	400	80	400	140
5	25	0.20	0.20	140	40	140	40
6	30	0.44	0.38	800	400	—	600
7	24	0.54	0.44	400	80	600	100
8	27	0.20	0.12	400	40	400	40
9	30	0.40	0.30	400	50	600	200
10	32	0.50	0.42	400	100	200	200

“—” indicates no measurable stereoscopic depth perception.
